# cGAS-STING triggers inflammaging-associated neurodegeneration

**DOI:** 10.1186/s13024-023-00666-9

**Published:** 2023-10-23

**Authors:** José M. Izquierdo

**Affiliations:** Centro de Biología Molecular ’Severo Ochoa’ (CBMSO), Consejo Superior de Investigaciones Científicas, Universidad Autónoma de Madrid (CSIC/UAM), C/ Nicolás Cabrera 1, Madrid, 28049 Spain

**Keywords:** Neurosenescence, Neuroinflammation, Neurodegeneration

In their recent article in Nature, Gulen et al. [1] established the cyclic GMP-AMP synthase (cGAS)-stimulator of interferon genes (STING) pathway as a regulatory driver of inflammaging-associated neurodegeneration in mice. Pharmacological blockade of this pathway in aged mice prevented inflammaging and improved cognitive and motor performance. In the same vein, chemical intervention attenuated a toxic murine model of cGAS gain-of-function in microglia leading to neuroinflammation, neurotoxicity and impaired memory capacity. These observations point to cGAS-STING as a targetable molecular sensor in human immunosenescence, aged-related inflammation and neurodegeneration.

Inflammation is an important defense against infection, but surprisingly little is known about immune system aging and whether it contributes to inflammation, and to what extent immunosenescence drives the low-grade inflammation associated with aging. The underlying causes of inflammation in this context remain unclear but several plausible models have been proposed. For example, the ‘Garb-aging’ theory [2] posits that damage to macromolecules and the progressive failure of repair and autophagy increases the level of cellular ‘garbage’, triggering innate immune signaling and inflammation [2]. This is compatible with identified hallmarks of aging including loss of proteostasis, mitochondrial dysfunction and genomic instability [3]. The strong links between inflammation, immunosenescence, frailty and age-related diseases are further highlighted by the development of an “aging clock” based on neuroinflammatory signatures [3].

**Fig. 1:**
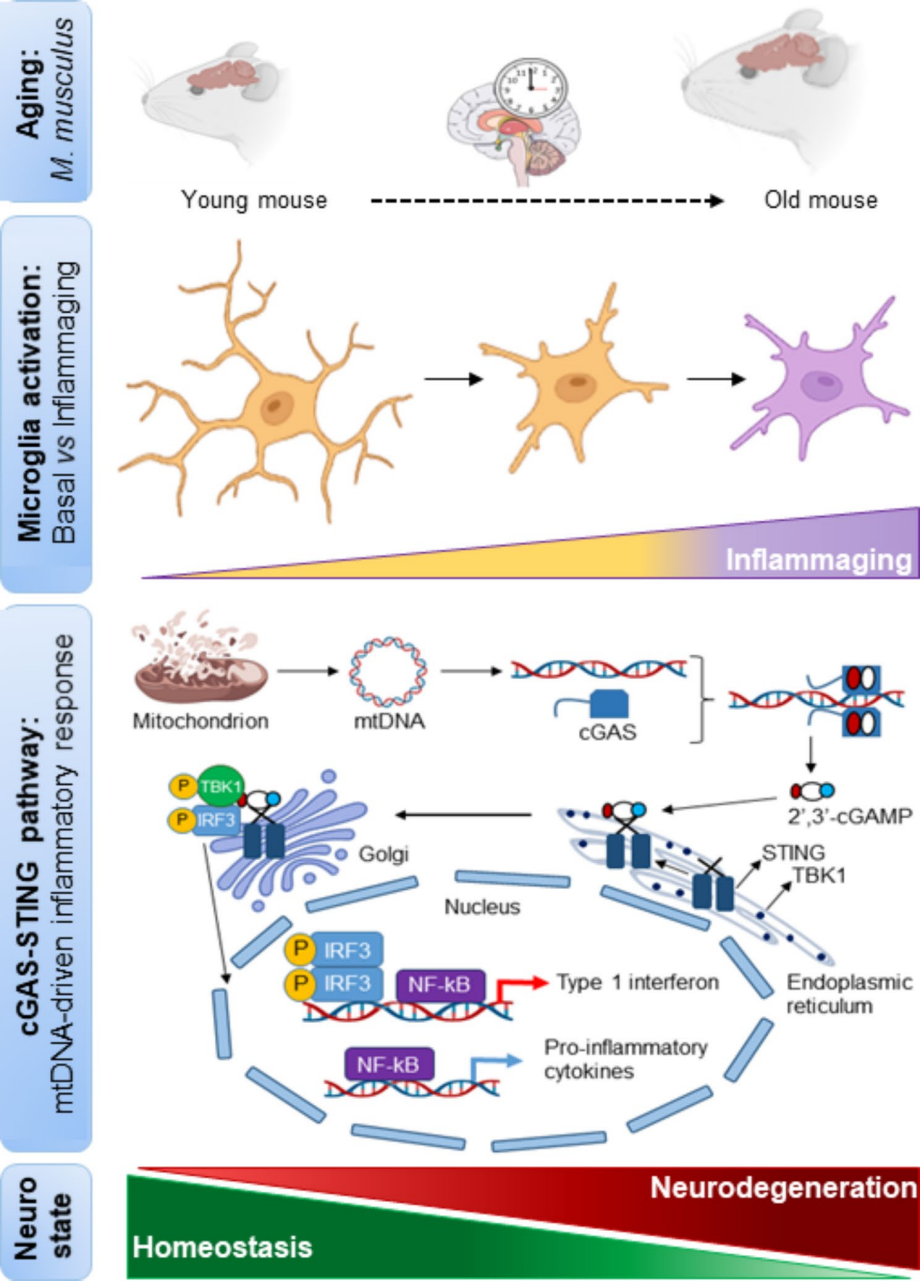
**cGAS-STING pathway leads to inflammation and microglia-dependent neurodegeneration.** Overview of murine brain aging, homeostatic and activated microglia, induction of mitochondrial DNA leakage-dependent cGAS-STING pathway, synthesis of type I IFN and proinflammatory cytokines during loss of homeostasis, leading to neurodegeneration

Inflammation is prominent in several neurodegenerative diseases [4], and there is evidence linking these pathologies to cGAS-STING dysregulation, which can disrupt cellular and organismal homeostasis by feeding aberrant innate immune responses [5, 6] (Fig. 1). Detection of foreign and self DNA is a crucial element of immunity, which in mammals is greatly aided by the cGAS-STING pathway, which couples DNA sensing to the induction of potent innate immune defenses independent of pathogen-specific features [6]. Binding of cGAS to DNA allosterically activates its catalytic function to produce 2′3′-cyclic GMP-AMP (cGAMP), a second messenger and potent STING agonist. Mechanistically, the cGAS-STING pathway transduces a signal from a broad repertoire of immunogenic DNA species to specific effector proteins, such as the transcription factors IRF and NF-kB, which alter the expression of proinflammatory genes, such as type 1 interferon (type 1 IFN) and inflammatory cytokines, thereby eliciting an immune response. However, evidence that dysregulation of this versatile innate immune sensing system can disrupt cellular and organismal homeostasis by feeding aberrant innate immune responses associated with various pathophysiological conditions is growing, such as neurodegeneration [5, 6] (Fig. 1).

In an outstanding paper published in Nature, Gulen et al. [1] investigated the cGAS-STING pathway in neuroinflammation and neurodegeneration associated with aging. They first reproduced and extended previous findings by observing that administration of H-151––a potent and covalent antagonist of STING [7]––inhibits cGAS-STING signaling in human senescent cells by selectively suppressing the induction of pro-inflammatory and interferon-stimulated genes. The authors next explored cGAS-STING signaling in the brain of aged (26-months-old) mice and investigated their tolerance to H-151. Aged mice exhibited a characteristic inflammatory signature associated with type 1 IFN induction in the kidney and liver. Compared with placebo-treated animals, H-151-treated mice exhibited diminished STING expression and the reduced expression of inflammatory genes and markers of kidney damage, findings that were validated in aged *Sting1*^*−/−*^ mice. They next assessed whether STING-dependent inhibition affected the physical and cognitive fitness of aged mice, finding a significant improvement in both abilities. STING thus acts as an important driver of inflammaging in central and peripheral nervous systems linked to cognitive decline and physical frailty.

To understand the contribution of cGAS-STING to brain aging, the authors next performed histological analyses in aged mice, which revealed prominent microgliosis that was dampened under STING inhibition. Likewise, STING blockade protected mice from neuronal loss in the hippocampus and increased local synaptophysin levels, overall demonstrating that STING influences brain homeostasis in aged mice. Finally, assessment of the brain intrinsic activity of cGAS-STING signaling in aged mice indicated that STING activation involved aberrant cGAS activity.

To discover the mechanisms underlying cGAS-STING activation in the brain, the authors performed bulk RNA sequencing analysis of the hippocampus of young (aged 8–10 weeks-old) and old (aged 19–20 weeks-old) mice treated with H-151 or vehicle, which identified many hundreds of differentially-regulated genes. Of these, a specific group were involved in innate immunity and included genes related to type 1 IFN signaling and microglia function, and their overexpression was attenuated in mice administered with H-151. This led the authors to consider that the microglia were the target of neurodegenerative effects of STING in brain of aged mice. Indeed, immunological analyses revealed that phosphorylated (active) STING was enriched in the hippocampi of old mice but not young mice, accompanied by the expression of type 1 IFN and pro-inflammatory genes.

To gain insight into the causes of cGAS-STING activation in microglia, the authors assessed mitochondrial DNA (mtDNA) as a central activator of cGAS-STING signaling [8]. Electron microscopy and expression analysis of microglia from aged mice revealed deformed mitochondria with changes in their internal ridge structure, suggesting possible breakage and release of mtDNA into the cytosol in the absence of genomic or nuclear DNA release. This was corroborated using high-resolution imaging showing cytosolic accumulation of mtDNA nucleoids adjacent to the outer membrane of mitochondria in microglia uniquely from old mice. Ex vivo and in vitro experiments confirmed the link between mtDNA and cGAS-STING signaling in both the IFN-dependent pro-inflammatory and inflammatory response and the senescence phenotype during aging.

To demonstrate a casual link between the cGAS-STING pathway and the neuropathological phenotype, the authors generated a toxic murine model of cGas gain-of-function: a conditional transgenic mouse (*Tmem119-creER*^*T2*^*-Cgas*^*WT/R241E*^) that expressed a mutant Cgas^R241E^ (corresponding to human CGAS^R255E^) that physiologically disrupts nucleosome binding after tamoxifen administration. Induced transgenic mice expressed Cgas^R241E^ mostly in microglia and brain macrophages, concomitant with a strong increase in the number of activated microglia cells in different brain regions together with expression of inflammatory and type 1 IFN-related genes, with no increase in inflammatory gene expression observed in the spleen; again H-151 administration attenuated microglial activation [1].

To understand the cGAS-controlled transcriptional program in microglia, the authors performed single-nucleus RNA-sequencing (snRNA-seq) analysis of microglia-enriched cells, which identified three distinct transcriptional states previously identified as disease-associated (DAM), IFN-associated and neurodegenerative microglial states, all related to neurodegenerative and aging conditions. These results were corroborated in microglia samples, establishing that in the absence of additional signals cGAS was sufficient to recapitulate microglial activation states associated with pathology and aging. Further transcriptomic analyses of microglia cells, astrocytes, oligodendrocytes and neurons revealed that cGAS activation in microglia had direct effects on the transcriptional programs of non-immune glia cells, resembling the changes that occur in aging brains.

Finally, to determine whether cGAS activity in microglia could initiate neurodegeneration, the authors examined two aspects related to neuronal function in the hippocampus: learning capacity and neuron density. Using Cgas^R241E^ transgenic mice, they observed that both parameters were impaired. Importantly, administration of H-151 attenuated the weakened learning ability, suggesting a link between STING-dependent pro-inflammatory activity and neuronal function. To dissect the neurotoxic processes underlying the gain of cGAS-STING activity in microglia, they co-cultured wild-type primary neurons and microglia from Cgas^R241E^ transgenic mice, finding that cGAS-mediated neurotoxicity could be recapitulated using conditioned medium from Cgas^R241E^-expressing cells, indicating that the detected neurotoxicity involved the secretion of soluble mediators. By analyzing microglia snRNA-seq datasets they detected *Tnf* upregulation in the IFN-associated microglia state in *Cgas*^*R241E*^ mice. Interestingly, the addition of neutralizing antibodies against TNF rescued *Cgas*^*R241E*^-induced neuronal death, whereas blockade of type 1 IFN signaling had no effect. These observations point to a critical role of TNF associated with aberrant cGAS activity in compromising neuronal survival [1] (Fig. 1).

The findings of Gulen et al. [1] have several implications and raise interesting questions. Future goals should include the following aspects: (i) is the cGAS-STING pathway activated during inflammation-associated human neurodegeneration?, (ii) could cGAS-STING activation be a good prognostic, diagnostic and disease progression/outcome marker for human inflammaging, immunosenescence and neurodegeneration?, (iii) could anti-inflammatory drugs target the cGAS-STING pathway in hyperactivated microglia to prevent aging/inflammation-associated neurodegeneration?, (iv) could mitochondrial dysfunction followed by mtDNA release be a good to target for inflammaging, immunosenescence and other aging-associated pathologies?, and (v) is the cGAS-STING pathway a primary target to alleviate other human diseases associated with inflammaging such as liver failure, muscular atrophy/dystrophy, obesity and neurological disorders?

The exciting paper by Gulen *et a*l [1] establishes the cGAS-STING pathway in murine inflammation, neuroinflammation and neurodegeneration, and represents a proof-of-concept for the role of microglia in inflammation-associated neurodegeneration. Their findings suggest that prevention of degeneration and neuronal death associated with aging might involve blocking and reprogramming sustained hyperactivation of microglia. In part, this could be achieved by the development of more specific anti-inflammatory drugs. It might also be beneficial to exploit the protective aspect of these cells to bolster their immune and regenerative roles as immunological gatekeepers of neurological health.
